# Vital Role of PINK1/Parkin-Mediated Mitophagy of Pulmonary Epithelial Cells in Severe Pneumonia Induced by IAV and Secondary *Staphylococcus aureus* Infection

**DOI:** 10.3390/ijms26094162

**Published:** 2025-04-27

**Authors:** Caiyun Huo, Yuli Li, Yuling Tang, Ruijing Su, Jiawei Xu, Hong Dong, Yanxin Hu, Hanchun Yang

**Affiliations:** 1 Key Laboratory of Animal Epidemiology of Ministry of Agriculture and Rural Affairs, National Key Laboratory of Veterinary Public Health and Safety, College of Veterinary Medicine, China Agricultural University, Beijing 100193, Chinadaisarlin@outlook.com (Y.L.); yulingtang1868@163.com (Y.T.); suruijingcomeon@126.com (R.S.); yanghanchun1@cau.edu.cn (H.Y.); 2 Beijing Key Laboratory of Traditional Chinese Veterinary Medicine, Beijing University of Agriculture, Beijing 102206, China; donghong523@163.com

**Keywords:** influenza A virus, *Staphylococcus aureus*, secondary infection, pulmonary epithelial cells, mitophagy

## Abstract

Influenza A virus (IAV) infection causes considerable morbidity and mortality worldwide, and the secondary bacterial infection further exacerbates the severity and fatality of the initial viral infection. Mitophagy plays an important role in host resistance to pathogen infection and immune response, while its role on pulmonary epithelial cells with viral and bacterial co-infection remains unclear. The present study reveals that the secondary *Staphylococcus aureus* infection significantly increased the viral and bacterial loads in human lung epithelial cells (A549) during the initial H1N1 infection. Meanwhile, the secondary *S. aureus* infection triggered more intense mitophagy in A549 cells by activating the PINK1/Parkin signaling pathway. Notably, mitophagy could contribute to the proliferation of pathogens in A549 cells via the inhibition of cell apoptosis. Furthermore, based on an influenza A viral and secondary bacterial infected mouse model, we showed that activation of mitophagy was conducive to the proliferation of virus and bacteria in the lungs, aggravated the inflammatory damage and severe pneumonia at the same time, and eventually decreased the survival rate. The results elucidated the effect and the related molecular mechanism of mitophagy in pulmonary epithelial cells following IAV and secondary *S. aureus* infection for the first time, which will provide valuable information for the pathogenesis of virus/bacteria interaction and new ideas for the treatment of severe pneumonia.

## 1. Introduction

Influenza A virus (IAV) causes respiratory infection in the form of seasonal flu and outbreaks, leading to rapid human-to-human transmissibility and high mortality [1]. Based on the report of the World Health Organization (WHO), epidemics of influenza cause 3 to 5 million severe cases annually, and among them, 290,000 to 650,000 patients die from respiratory diseases globally. Meanwhile, secondary bacterial infections are the most common complications of primary IAV infection, with high morbidity and mortality reported during both seasonal influenza epidemics and pandemics. *Streptococcus pneumoniae* and *Staphylococcus aureus* (*S. aureus*) are two of the most commonly recognized pathogens causing secondary infections. During the most devasting IAV pandemic, the 1918 Spanish Flu, more than 40 million patients died that were accompanied by a secondary *Streptococcus* infection [2]. During the 2009 pandemic, despite the widespread use of antibiotics, 25 to 40% of deaths were attributed to bacterial co-infections [3,4]. *S. aureus* is a symbiotic organism that exists in many healthy people and can cause a series of diseases, such as endocarditis and necrotizing pneumonia [5]. In recent years, the emergence of highly virulent community-related methicillin-resistant *Staphylococcus aureus* (CA-MRSA) has made *S. aureus* the main reason for pneumonia in U.S. hospitals. The role of immune disorders in co-infection has been extensively studied and is considered one of the main potential causes of increased bacterial susceptibility after primary influenza infection. Therefore, IAV and secondary *S. aureus* infections pose a great threat to public health, and related research on the pathogenesis mechanism is of great significance.

Mitochondria are the organelles of eukaryotic cells that produce energy, which are encased in a unique bilayer membrane where energy from the organ is converted into ATP through oxidative phosphorylation to support cell survival [6]. The oxidative phosphorylation process produces a large number of reactive oxygen species (ROS), which can lead to mitochondrial damage. If ROS is not repaired or removed in time, the damaged mitochondria accumulate within the cells, increasing the cell burden and leading to the loss of cell function. Mitophagy is the process by which autophagosomes selectively engulf damaged mitochondria and are subsequently degraded by lysosomes [7]. Therefore, mitophagy is important for the degradation of the excess damaged mitochondria to maintain cellular homeostasis and survival. Nowadays, there is accumulating evidence indicating that viruses can promote the replication and proliferation in host cells through hijacking mitophagy mechanisms, including hepatitis C virus, human immunodeficiency virus, and respiratory syncytial virus [8,9,10]. Virus-induced mitophagy leads to the degradation of the antiviral signaling protein MAVS localized in the outer mitochondrial membrane (OMM), thereby inhibiting the host immune signaling. In recent years, great efforts have been made to further understand the association between virus-induced mitochondrial autophagy and host immunity. In bacteria, studies have shown that certain pathogens can exploit mitophagy to enhance their intracellular survival. For example, *S. aureus* has been reported to induce mitophagy in bovine mammary epithelial cells through activation of the p38–PINK1–Parkin signaling pathway, thereby promoting its persistence within host cells [11].

To date, the relationship between mitophagy and viral/bacterial secondary infection has been poorly reported. Herein, to further explore the relationship between mitochondria and secondary infections as well as the pathogenesis mechanism and facilitate our understanding of the process of IAV and secondary *S. aureus* infection, we selected the representative bacteria—highly virulent MRSA—and aimed to investigate the occurrence and roles of mitophagy in pulmonary epithelial cells following IAV and secondary *S. aureus* infection.

## 2. Results

### 2.1. IAV and Secondary S. aureus Infection Reduce the Viability of Pulmonary Epithelial Cells and Promote the Intracellular Proliferation of Viruses and Bacteria

**Figure 1:**
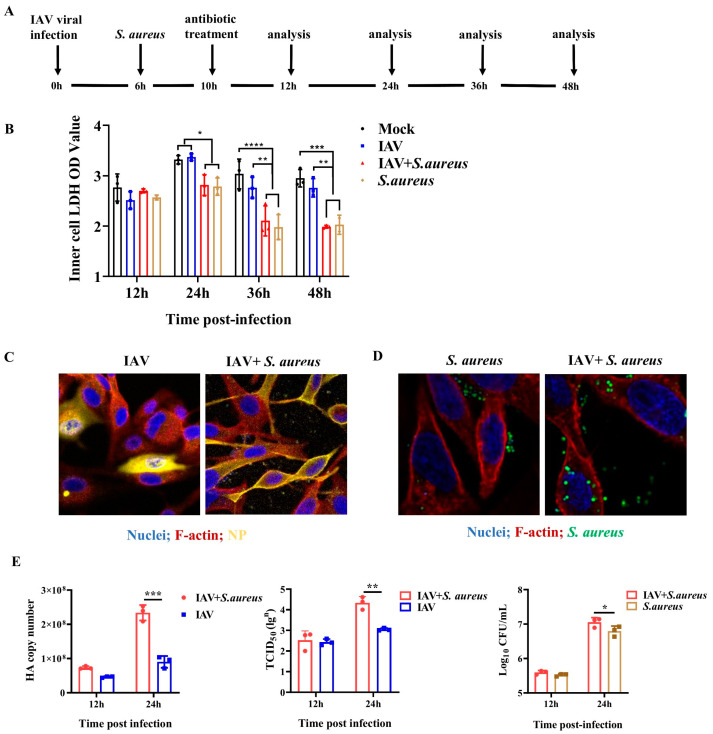
Effects of IAV and secondary *S. aureus* infection on the viability of A549 cells as well as the intracellular proliferation of virus and bacteria. (**A**) Schematic diagram of the experimental protocols utilized in this study. A549 cells were infected with the H1N1 virus (MOI = 0.1) at 0 h and *S. aureus* (1 CFU/cell) at 6 h and subsequently were treated with antibiotics at 10 h. Then, samples were collected at 12 h, 24 h, 36 h, and 48 h, respectively (*n* = 3). (**B**) LDH activity was assessed by the Lactic Dehydrogenase Release Assay Kit. (**C**) Viral NP protein expression was measured by immunofluorescence staining. (**D**) The invaded bacteria of the infected A549 cells by immunofluorescence staining. (**E**) Viral HA gene expression, viral titers, and bacterial proliferation in A549 cells were measured by RT-qPCR, TCID50 assay, and CFUs plate counting, respectively. Statistical significance was tested using a two-way analysis of variance (ANOVA) among groups. * *p* < 0.05; ** *p* < 0.01; *** *p* < 0.001; **** *p* < 0.0001.

To examine the influence of co-infection on pulmonary epithelial cells in vitro, an established A549 cell line model was used, as shown in Figure 1A. In this model, A549 cells were initially infected with the H1N1 virus (MOI = 0.1) for 6 h and sequentially infected with *S. aureus* (MOI = 1) for 4 h. Under TEM, IAV and *S. aureus* particles could be found in A549 cells at 24 h post-infection, indicating the successful establishment of the co-infection cell model (Figure S1). Membrane damage occurs when cells are infected by pathogenic microorganisms. Here, we also investigated the damage of IAV and secondary *S. aureus* infection to the pulmonary epithelial cells using the LDH activity test. As seen in Figure 1B, co-infection could significantly inhibit the cellular viability of A549 cells compared with the single IAV infection at 24 h, 36 h, and 48 h, especially at 36 h and 48 h.

To determine whether co-infection affects the proliferation of IAV and *S. aureus*, the viral NP protein expression and invaded bacteria of the infected A549 cells were measured. From Figure 1C,D, we found that viral NP protein expression and invaded bacteria in co-infected cells were higher than those infected with IAV or *S. aureus* alone. Meanwhile, RT-qPCR, TCID_50_ assay, and CFUs plate counting also confirmed that co-infection was able to increase the viral HA gene expression, viral titers, and bacterial proliferation in A549 cells; the significance could be shown at 24 h post-infection (*p* = 0.0354) (Figure 1E). Taken together, these results suggest that IAV and secondary *S. aureus* infection can reduce the viability of pulmonary epithelial cells and promote the intracellular proliferation of viruses and bacteria.

### 2.2. IAV and Secondary S. aureus Infection Cause the Mitochondria Damage in Pulmonary Epithelial Cells

**Figure 2:**
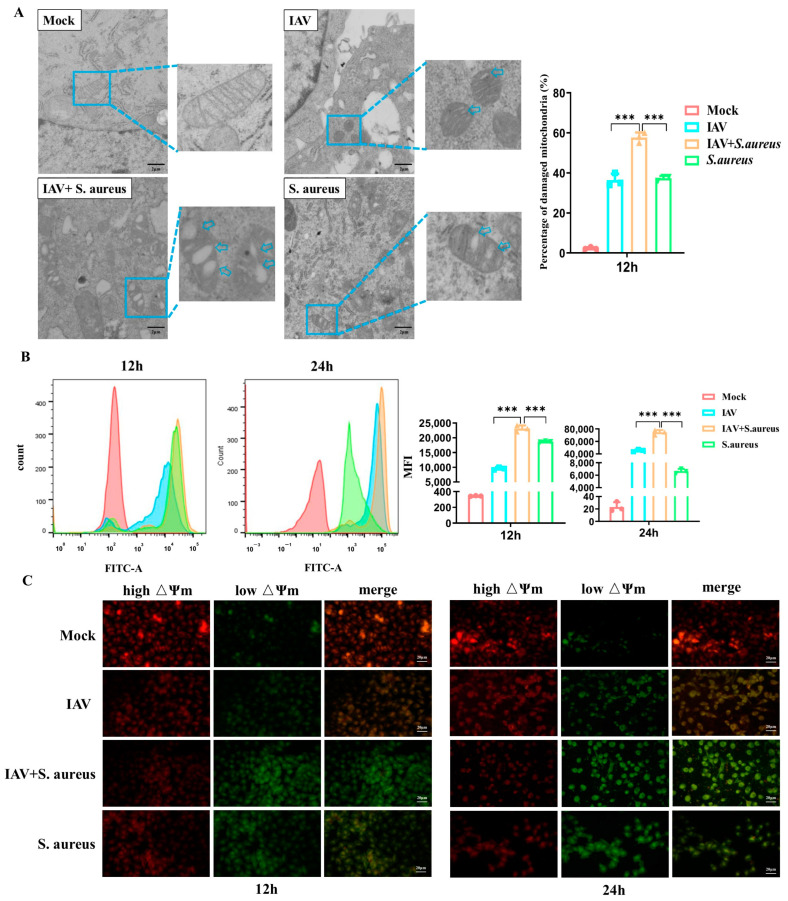
IAV and secondary *S. aureus* infection caused the mitochondria damage in A549 cells. (**A**) Changes in mitochondrial morphology in infected A549 cells were detected by TEM. (**B**) The levels of ROS in infected A549 cells were measured by Reactive Oxygen Species Assay Kit (*n* = 3). (**C**) A549 cells treated as above were labeled with fluorescent probe JC-1 to evaluate MMP changes in situ by confocal microscopy. J-aggregate (high △Ψm): red; JC-1 monomer (low △Ψm): Green. *** *p* < 0.001.

Subsequently, we detected the mitochondria damage caused by co-infection in A549 cells. As displayed in Figure 2A, obvious morphological changes occurred in co-infected A549 cells compared to those cells infected with virus or bacteria alone, including severe mitochondrial swelling, broken cristae, and disappearance.

In order to verify whether the mitochondria function was altered, we measured the production of ROS and mitochondrial membrane potential (MMP, △Ψm) in A549 cells infected with IAV and secondary *S. aureus*. As shown in Figure 2B, the levels of ROS produced in co-infected A549 cells were also much higher than those in cells infected with virus or bacteria alone at 12 h and 24 h post-infection, respectively. Based on JC-1 staining, a visible decrease in the red fluorescence and elevation of the green fluorescence were observed by confocal microscopy in co-infected A549 cells compared with the cells infected by a single pathogen (Figure 2C). Together, the results demonstrate that IAV and secondary *S. aureus* infection cause much more severe mitochondrial damage in pulmonary epithelial cells.

### 2.3. IAV and Secondary S. aureus Infection Induce the PINK1/Parkin-Mediated Mitophagy in Pulmonary Epithelial Cells

**Figure 3:**
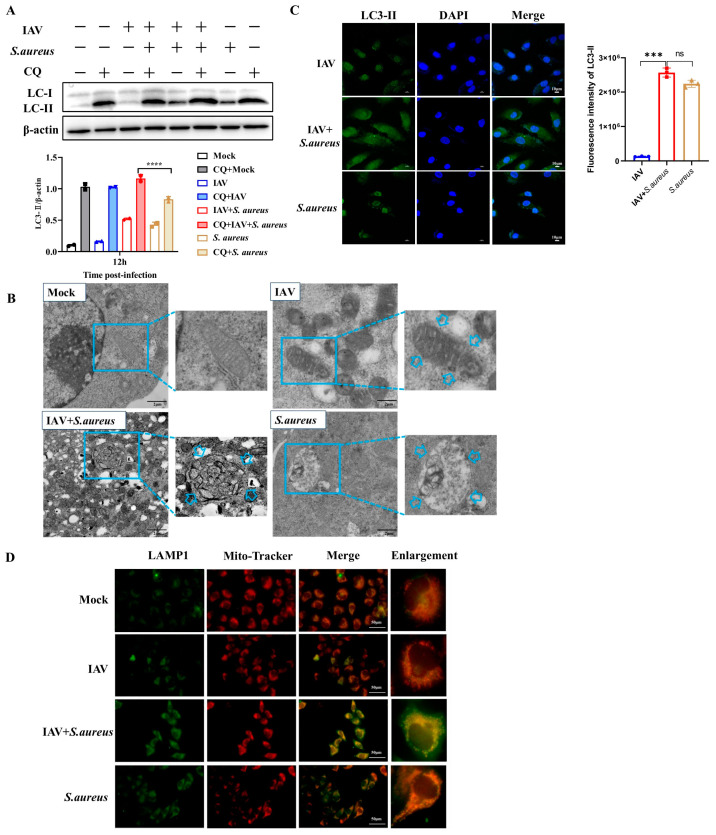
IAV and secondary *S. aureus* infection induce mitophagy in A549 cells. (**A**,**B**) The autophagosome signature protein of LC3 in infected A549 cells was detected by Western blot and LC3 immunostaining, respectively. Arrows indicated the formation of mitophagosomes. (**C**) The bilayer-membrane mitochondria were observed in infected A549 cells under the TEM. (**D**) Immunofluorescence detected the colocalization (in yellow) of mitochondria (in red) and lysosomes (in green) in A549 cells. Colocalization analysis of the different channels in “Merge”. Statistical significance was tested using a two-way analysis of variance (ANOVA) among groups. *** *p* < 0.001; **** *p* < 0.0001. ns: no significance.

As obvious severe mitochondria damage was observed in the co-infected A549 cells, we aimed to determine the occurrence of mitophagy. Firstly, the autophagosome signature protein of microtubule-associated-proteinlight-chain-3 (LC3) in A549 cells was detected by Western blot. As shown in Figure 3A,B, co-infection could extremely increase the level of LC3-II in cells compared to that in the control group or the single pathogen group. Subsequently, chloroquine (CQ) was applied to inhibit the fusion of autophagosomes and lysosomes for the detection of the autophagic flux. It seemed that the secondary *S. aureus* infection significantly induced the smooth autophagic flux and led to the accumulation of mitochondrial autophagosomes. Moreover, the bilayer-membrane mitochondria were observed in co-infected A549 cells under TEM, indicating the formation of mitophagosomes and the activation of mitophagy (Figure 3C). Furthermore, immunofluorescence staining was performed to investigate the colocalization of mitochondria with the lysosome signature protein LAMP1. We found that secondary *S. aureus* infection could facilitate the fusion of mitophagosomes with lysosomes (Figure 3D). Together, these results suggest that IAV and secondary *S. aureus* infection can induce mitophagy in pulmonary epithelial cells.

**Figure 4:**
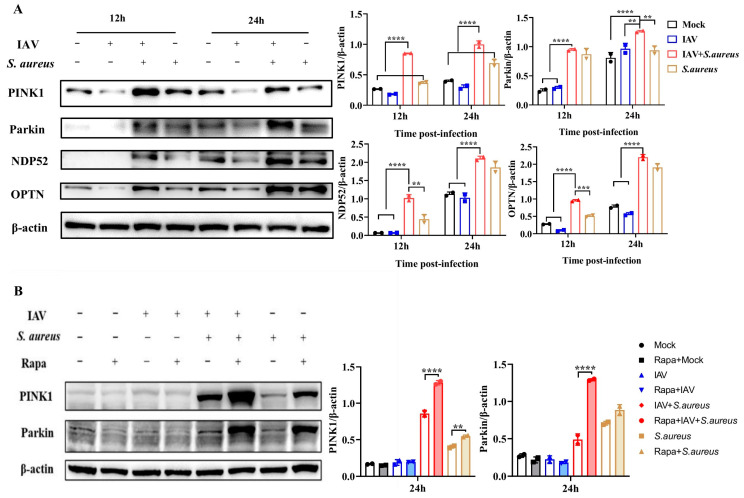
Mitophagy in A549 cells with IAV and secondary *S. aureus* infection was mediated by PINK1/Parkin. (**A**) The protein expressions of PINK1, Parkin, OPTN, and NDP52 were detected by Western blot (*n* = 2). (**B**) The Rapa (mTORC1 inhibitor) at 10 μM was added into cells for 24 h; then, the p-mTOR, LC3, PINK1, and Parkin were detected by Western blot (*n* = 3). Statistical significance was tested using a two-way analysis of variance (ANOVA) among groups. ** *p* < 0.01; *** *p* < 0.001; **** *p* < 0.0001.

As the occurrence of mitophagy in A549 cells with IAV and secondary *S. aureus* infection, the related molecular mechanism was deeply investigated. PINK1, a Ser/Thr kinase in the cytoplasm, can be accumulated at the outer mitochondrial membrane and activated through auto-phosphorylation when the mitochondria are damaged [12]. It is capable of acting on the upstream of Parkin, regulating the mitophagy and co-degrading the damaged mitochondria [13]. Thus, PINK1 and Parkin maintain a stable mitochondrial network by promoting autophagy to eliminate damaged mitochondria. As mentioned above, MMP significantly declined in A549 cells infected with IAV and secondary *S. aureus*. Interestingly, previous research has proved that MMP is critical in PINK1/Parkin-mediated mitophagy [14]. Therefore, we investigated whether the mitophagy in A549 cells with IAV and secondary *S. aureus* infection was mediated by PINK1/Parkin. As shown in Figure 4A, the protein expressions of PINK1 and Parkin in A549 cells infected with secondary *S. aureus* were much higher than those in the control group or the single pathogen group at 12 h and 24 h post-infection, respectively. Meanwhile, the adaptor proteins of OPTN and NDP52 were also significantly up-regulated in co-infected A549 cells, indicating the involvement of mitophagy. Rapamycin complex 1 (mTORC1) has been validated to specifically regulate the autophagic clearance of damaged mitochondria [15]. Here, we added rapamycin (Rapa), an inhibitor of mTORC1, into cells and found that the levels of PINK1 and Parkin induced by secondary *S. aureus* infection were further improved (Figure 4B). Thus, these results illustrate that IAV and secondary *S. aureus* infection can induce PINK1/Parkin-mediated mitophagy.

### 2.4. Mitophagy Facilitates Pathogen Proliferation in Pulmonary Epithelial Cells with IAV and Secondary S. aureus Infection

**Figure 5:**
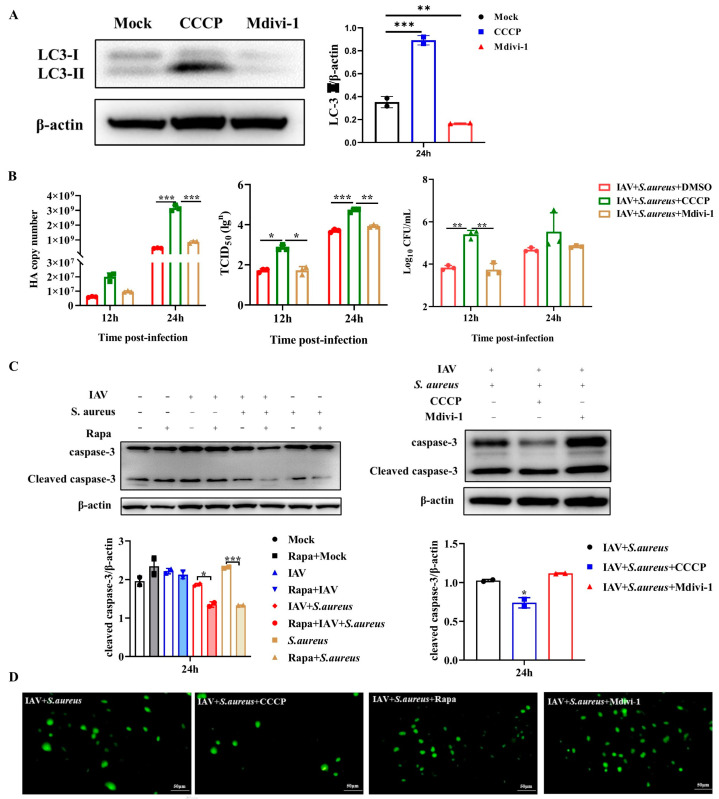
Effects of mitophagy on both viral and bacterial proliferation in A549 cells. Cells were treated with CCCP (mitophagy activator), Mdivi-1 (mitophagy inhibitor), and Rapa (mTORC1 inhibitor) at 10 μM for 24 h, respectively. (**A**) The autophagosome signature protein of LC3 in infected A549 cells was detected by Western blot (*n* = 2). (**B**) Viral HA gene expression, viral titers, and bacterial proliferation in A549 cells were measured by RT-qPCR, TCID50 assay, and CFUs plate counting, respectively (*n* = 3). (**C**) The protein level of caspase-3 and cleaved caspase-3 in infected A549 cells was detected by Western blot (*n* = 2). (**D**) The TUNEL assay was used to measure apoptosis in infected A549 cells. Green showed positive TUNEL signals. Statistical significance was tested using a two-way analysis of variance (ANOVA) among groups. * *p* < 0.05; ** *p* < 0.01; *** *p* < 0.001.

Previous studies have shown that a variety of viruses, such as hepatitis C virus and human immunodeficiency virus, can maintain persistent infection and suppress the innate immune response by promoting mitochondrial fission and inducing mitophagy [8,16]. Hence, the effect of mitophagy on intracellular pathogen proliferation during secondary infection was further investigated. Firstly, the cells were treated with CCCP (a specific activator of PINK1/Parkin-mediated mitophagy) and Mdivi-1 (a specific inhibitor of PINK1/Parkin-mediated mitophagy) at an optimal dose of 10 μM (Figure S2) for 24 h to assess their effects on LC3-II expression in A549 cells, respectively. As shown in Figure 5A, CCCP treatment significantly increased LC3-II expression in A549 cells, whereas Mdivi-1 treatment showed a significant inhibition of LC3-II expression. Subsequently, these treated cells were infected with IAV and secondary *S. aureus* or alone. At 12 h and 24 h post-infection, CCCP pre-treatment significantly increased the expression of the intracellular viral HA gene, viral titers, and CFUs of intracellular bacteria (Figure 5B). In contrast, Mdivi-1 pre-treatment had dramatic inhibitory effects on viral HA gene expression and bacterial CFUs. Above all, mitophagy facilitates the proliferation of virus and bacteria in A549 cells infected with IAV and secondary *S. aureus*.

It has been proved that mitophagy can suppress apoptosis to promote virus proliferation [17]. Here, the effect of mitophagy on apoptosis was also evaluated in A549 cells infected with IAV and secondary *S. aureus*. As displayed in Figure 5C, Rapa and CCCP treatment decreased the level of caspase-3 in cells infected with secondary *S. aureus* infection. Meanwhile, Mdivi-1 treatment increased the cleavage level of caspase-3 in co-infected cells. To further confirm the occurrence of apoptotic responses, in situ TUNEL, an assay that detects DNA strand breakage, which is a hallmark of apoptosis, was performed. Positive signals of apoptosis were detected in IAV + *S. aureus* + Rapa and IAV + *S. aureus* + Mdivi-1 groups rather than in mock-treated cells, which were consistent with the result of caspase-3 levels (Figure 5D). Thus, these results demonstrate the inhibitory effect of mitophagy on apoptosis in A549 cells following secondary *S. aureus* infection, which could also explain the reason behind the pathogen proliferation after mitophagy occurs.

### 2.5. Mitophagy Exacerbates the Lung Injury in Mice with IAV and Secondary S. aureus Infection

**Figure 6:**
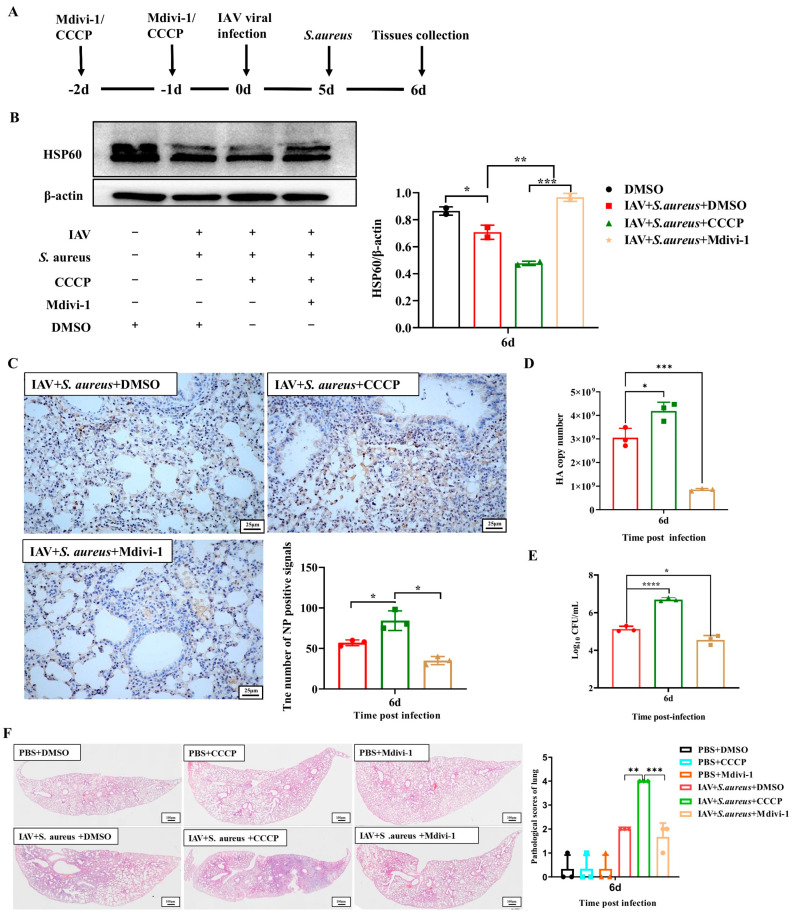
Effects of mitophagy on the lung injury in mice following IAV and secondary *S. aureus* infection. (**A**) Mice were abdominally injected with CCCP (5 mg/kg) and Mdivi-1 (25 mg/kg) for two days before infection. After infection of IAV and secondary *S. aureus*, lung tissues were collected at day 6 (*n* = 10). (**B**) The protein level of HSP60 was detected by Western blot (*n* = 3). (**C**) The expression of viral NP at day 6 post-infection with IHC staining and scored by an examiner blinded to the study (*n* = 2). (**D**) The HA copy numbers in the lung of mice at day 6 post-infection with RT-qPCR (*n* = 3). (**E**) Bacterial proliferation in the lung of mice was measured by CFUs plate counting (*n* = 3). (**F**) Lung pathology at day 6 post-infection with H&E staining. Statistical significance was tested using a two-way analysis of variance (ANOVA) among groups. * *p* < 0.05; ** *p* < 0.01; *** *p* < 0.001; **** *p* < 0.0001.

As mentioned above, the activation of mitophagy can promote viral and bacterial proliferation within pulmonary epithelial cells infected with IAV and secondary *S. aureus*. To further investigate the effect of mitophagy on severe pneumonia in mice during secondary *S. aureus* infection, the mice were treated with CCCP and Mdivi-1, respectively. At first, their influences on mitophagy were validated by the detection of HSP60 in lung tissue. As seen in Figure 6A,B, the CCCP treatment significantly reduced the expression of HSP60 compared with the DMSO treatment, while the Mdivi-1 treatment significantly improved. Therefore, CCCP could activate the mitophagy in the lungs of mice, and Mdivi-1 inhibited the mitophagy. Moreover, the levels of NP protein, HA gene, and CFUs were significantly increased in the CCCP group and decreased in the Mdivi-1 group. These findings were in accordance with the observations in vitro (Figure 6C–E).

Subsequently, mice pre-treated with CCCP and Mdivi-1 were infected with IAV and secondary *S. aureus* or alone, then the clinical symptoms and pathological changes in the lungs were assessed. As shown in Figure S2A, mice in the CCCP treatment group had more severe symptoms than those in the DMSO group, which were characterized by mental malaise, erect dorsal hairs, arched back, squinting, and curling. Nevertheless, mice in the Mdivi-1 treatment group had smoother back hairs and better mental conditions. In the lungs, co-infected mice that were pre-treated with CCCP had more severe symptoms compared with those pre-treated with DMSO, such as swollen, enlarged hemorrhage area and wet surface (Figure S2B). However, the symptoms in the lungs of mice in the secondary infection group administered with Mdivi-1 were alleviated, which displayed pink lobes and reduced hemorrhage (Figure S3A,B). Further histopathological observations showed that the CCCP administration aggravated the lesions of lung tissue in the secondary infected mice, and the area of interstitial inflammation was increased, while Mdivi-1 administration significantly reduced the inflammatory response (Figure 6F). Above all, inhibition of mitophagy has an effective protection effect against severe pneumonia induced by secondary infection in mice.

## 3. Discussion

Influenza and secondary bacterial infection are associated with IAV-related death, among which *Streptococcus pneumoniae* and *S. aureus* are the most commonly detected bacterial pathogens [18]. Nowadays, the detection rate of community-acquired MRSA has gradually increased, causing more severe consequences following IAV infection. However, the pathogenesis of secondary *S. aureus* infection following IAV infection still needs to be further explored.

The lung is the main target organ following influenza/bacteria co-infection. Once pathogens invade the pulmonary epithelial cells, the innate immune response is quickly activated to facilitate the elimination of pathogens and host survival. However, pathogens also evolve various strategies to enable themselves to remain infective and weaken the immune response of the host, thereby promoting their survival. Mitochondria are involved in a wide variety of physiological processes in cells, such as the regulation of the innate immune system as a signaling platform [19]. Dysregulation of mitochondrial activity leads to a decrease in ATP production and an increase in ROS production [20]. As a result, some viruses can disrupt the balance of the mitochondrial network and affect the metabolism and physiology of host cells to promote the immune escape [21]. In recent years, the function of mitophagy during multiple viral infections has been demonstrated, and viruses have developed different strategies to disrupt mitochondria and benefit from mitophagy. Viruses can induce intracellular signaling that triggers mitochondrial dysfunction and promotes PRKN-dependent or receptor-mediated mitophagy or directly trigger mitochondrial phagocytosis through their own viral components. For example, the M2 protein of IAV interacts with MAVS on the mitochondria and positively regulates MAVS-mediated innate immunity [22]. In addition, the M2 induces ROS production, which is necessary to activate autophagy and enhances the MAVS signaling pathway [22]. In some cases, virus can affect the steps of the mitochondrial cycle to be persistently infective and cause diseases. Herein, our study also aims to explore the occurrence of mitophagy in pulmonary epithelial cells during co-infection and its role in the pathogenic processes.

In our study, the in vitro model of pulmonary epithelial cells that was infected with IAV and secondary *S. aureus* was established. Although *S. aureus* is not traditionally classified as an intracellular pathogen, it is capable of being internalized by epithelial cells. The processes of bacterial adhesion and invasion involve complex interactions between bacterial surface components and host cell receptors, as well as the modulation of intracellular signaling pathways, including actin cytoskeleton rearrangement. It was found that the secondary infection enhanced the proliferation of IAV and *S. aureus* compared with single pathogen infection (Figure 1), which agreed with a previous study [23]. During the secondary infection, the influenza virus can promote bacterial susceptibility in various ways. For instance, the inhibition of innate T helper type 17 immunity induced by IFN can enhance the susceptibility of secondary bacterial pneumonia [24]. The antiviral activity of IFN can directly suppress the secretion of chemokines and acute pro-inflammatory cytokines by damaging the natural killer cells, further inhibiting the normal phagocytic activity of macrophages and neutrophils and eventually resulting in increased bacterial invasion [25]. Most of the mechanisms described above focus on the viral contribution to subsequent bacterial re-infection, but factors induced through bacterial co-infection may also affect the virus. It has been proposed that the bacterial protease of *S. aureus* is capable of cleaving the nascent hemagglutinin from the basal state into a fusion-active complex, which increases the viral titers and spread [26]. In our study, we illustrate that IAV and secondary *S. aureus* infection can induce mitophagy in the pulmonary epithelial cells to inhibit apoptosis and, thus, promote the intercellular survival of bacteria and viruses, which leads to disease aggravation.

Here, we also discovered a decline in MMP and an increase in ROS during the secondary infection (Figure 2). Studies have shown that MMP is necessary for MAVS-mediated antiviral signaling, and its decrease is associated with reduced antiviral response [27]. When mitochondria lose their membrane potential, PINK1 accumulates on the outer mitochondrial membrane and can recruit PRKN into the damaged mitochondria. PRKN binds more ubiquitin chains to mitochondria, promoting the fusion of autophagosomes with lysosomes [28]. ROS has been reported to act on the upstream of the PINK1/Parkin pathway, mediating mitochondrial autophagy. During IAV and secondary *S. aureus* infection, we demonstrated that secondary *S. aureus* infection could facilitate the formation of mitophagosomes and fusion of mitophagosomes to lysosomes (Figure 3). Moreover, autophagosomes enveloped by the biolayers membrane were also observed by TEM. Therefore, mitophagy can occur in pulmonary epithelial cells following IAV and secondary *S. aureus* infections. A previous study has proven that the interaction between IAV PB1-F2 and TUFM (Tu transcriptional elongation factor, mitochondria) is essential for MAVS degradation [29]. To our knowledge, this study on the occurrence of mitophagy triggered by bacteria secondary to viral infection will enrich the understanding of the pathogenic mechanisms of secondary infections.

According to the dependence on PRKN, the E3 ligase, the mitophagy mechanisms of mammalian cells can be divided into two categories: PRKN-dependent and non-PRKN-dependent mitophagy [30]. PRKN-dependent mitophagy is generally associated with alterations of MMP, leading to the mitochondrial depolarization that two key gene products are involved in: PINK1 and PRKN [28]. The ubiquitin and ubiquitin-like domains of Parkin can be recruited and phosphorylated by the activated PINK1, which can induce the activation of Parkin and transport it from the cytoplasm to the damaged mitochondria, thereby promoting the mitophagy in the end. The phosphorylated ubiquitin can also recruit autophagic receptors, such as OPTN and NDP52, to promote the clearance of damaged mitochondria [31]. Conversely, mitophagy that does not depend on PRKN usually involves the mitophagy receptors [7]. Several mitophagy receptors have been found in mammalian cells, including BCL2L13, BNIP3L/NIX, and FUNDC1 [29,32,33]. These mitophagy receptors are located on the outer membrane of the mitochondria and interact with LC3 through the LC3 interaction zone motif, promoting the autophagy system to engulf the mitochondria. Evidence has also suggested that there is an interaction between PRKN-dependent and PRKN-independent mitophagy effectors. For example, BNIP3L/NIX promotes CCCP-induced mitochondrial depolarization and also increases the mitochondrial priming effects by controlling the mitochondrial translocation of PRKN [34]. In this study, PINK1 and Parkin were significantly up-regulated at 12 h and 24 h following IAV and secondary *S. aureus* infection, and the expressions of adapter proteins of OPTN and NDP52 were also extremely promoted (Figure 4). These results indicate the occurrence of PRKN-dependent mitophagy in the secondary infection. Notably, the role of the receptor-mediated mitophagy on this secondary infection cannot be denied and is worthy of further exploring.

Despite the fact that the IAV and secondary *S. aureus* infection induced PRKN-dependent mitophagy in cells, the influence of this kind of mitophagy on the pathogenesis of secondary infections was unknown. *Listeria monocytogenes* promotes the binding of the LC3-interacting region (LIR) motif to LC3 by inducing oligomerization of the mitochondrial targeting sequence NLRX1, thereby inducing mitophagy and promoting bacterial survival [35]. Hepatitis C virus induces Parkin-mediated selective mitochondrial phagocytosis, and the silence of Parkin and PINK1 can inhibit the replication of HCV, suggesting that mitophagy may be beneficial for viral replication [36]. Here, we also confirmed that the activation of mitophagy by IAV and secondary *S. aureus* infection could contribute to the proliferation of bacteria and viruses in vitro (Figure 5). This finding was consistent with many previous studies, indicating that pathogens can hijack cell homeostasis to promote their survival. Previous research has explained the crosstalk between mitophagy and cell apoptosis. For instance, hepatitis B virus-induced mitochondrial dynamics protect virus-infected hepatocytes from apoptotic cell death to facilitate persistent virus infection [37]. Furthermore, we also detected changes in cell apoptosis following co-infection in vitro (Figure 5). The results showed that the expressions of the apoptotic protein caspase3 and caspase6 were all decreased as the activation of mitophagy or promoted as the inhibition of mitophagy. Thus, the activation of mitophagy inhibits cell apoptosis, which can explain the reason that mitophagy activation contributes to the survival of intracellular pathogens.

Consistent with the in vitro results, in the mouse model with the secondary infection, we also confirmed that the occurrence of mitophagy could significantly increase the bacterial and viral loads in the lungs of mice (Figure 6). Furthermore, the inflammatory damages in the lungs were also aggravated by the activation of mitophagy. This phenomenon of mitophagy that exacerbated the inflammatory response was consistent with previous studies on the relationship between mitophagy and immune response. Mitophagy can exacerbate the diseases by inhibiting the overactivation of NLRP3 inflammasomes and subsequent immune responses, thereby inhibiting the downstream immune signals against microbial infections and intracellular damage [38].

**Figure 7:**
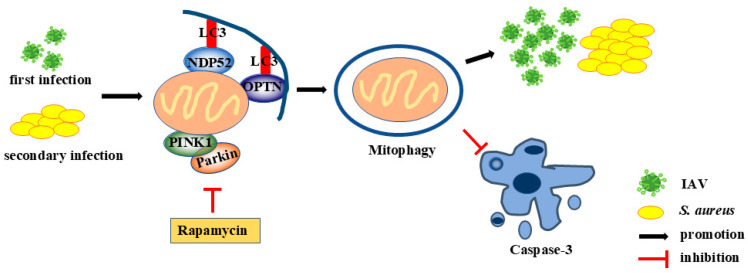
Overview of our findings.

In summary, for the first time, we provide new evidence that IAV and secondary *S. aureus* infection can activate PINK1/Parkin-mediated mitophagy in pulmonary epithelial cells, thus promoting the survival and replication of pathogens within target cells as well as inhibiting the cell apoptosis (Figure 7). Meanwhile, the activation of IAV and secondary *S. aureus* infection can also contribute to the proliferation of viruses and bacteria in the lungs of mice and aggravate the inflammatory damages. The findings provide a novel mechanism for viral and bacterial co-infection. These results may facilitate the development of new drugs and strategies to fight viral and secondary bacterial infections.

## 4. Materials and Methods

### 4.1. Reagents and Antibodies

Antibodies against the following proteins were used: anti-β-actin monoclonal antibody was purchased from Solarbio, Beijing, China. Parkin rabbit polyclonal antibody, PINK1 rabbit polyclonal antibody, HSP60 mouse monoclonal antibody, LAMP1 rabbit polyclonal antibody, Alexa Fluor 488-labeled goat-anti rabbit IgG, HRP-labeled goat-anti rabbit IgG and HRP-labeled goat-anti mouse IgG were purchased from Beyotime, Beijing, China. LC3A/B (D3U4C) XP^®^ rabbit mAb and caspase-6 antibody were purchased from CST, Danvers, Massachusetts, USA. Anti-SQSTM1/p62 antibody and anti-influenza A virus nucleoprotein antibody were purchased from Abcam, Cambridge, UK. OPTN rabbit polyclonal antibody and CALCOCO2 rabbit polyclonal antibody were purchased from Proteintech, Wuhan, Hubei, China. Caspase-3 was purchased from Abmart, Shanghai, China.

### 4.2. Virus, Bacterial Strains, and Cell Lines

The H1N1 (A/WSN/33) virus was provided by Dr. George F. Gao of the Institute of Microbiology, CAS, China, and the working stocks were generated in Madin–Darby canine kidney cells (MDCK) [39]. Virus titers were determined by standard plaque assay as previously described [39]. Methicillin-resistant *S. aureus* MRSA.T144 and ATCC 29,213 were kindly provided by Professor Kui Zhu, China Agricultural University, China. *S. aureus* was grown in brain heart infusion (BHI) broth (Land Bridge, Jiangsu, Nanjing, China) with constant shaking or on BHI agar plates (Land Bridge) overnight at 37 °C.

The human lung adenocarcinoma cell line A549 and MDCK cells were provided by the Cell Resource Center of the Peking Union Medical College (Beijing, China). The cells were cultured in Dulbecco’s modified Eagle’s medium (DMEM; HyClone, Logan, Utah, USA) containing 10% fetal bovine serum (FBS; HyClone), 100 U/mL penicillin and 100 μg/mL streptomycin.

### 4.3. Infection of Cells and Transmission Electron Microscopy (TEM) In Vitro

For in vitro infection experiments, A549 cells were seeded at 1 × 10^6^ cells/mL in a 6-well plate. Firstly, A549 cells were inoculated with H1N1 virus (MOI = 0.1) for 1 h at 37 °C. After washing with PBS 3 times, DMEM supplemented with 1% bovine serum albumin was added to each well for 6 h at 37 °C. Then, A549 cells were inoculated with 1 CFU/cell *S. aureus* (1 mL/well) for a further 4 h and inoculated with gentamicin (dissolved in DMEM, 200 μg/mL) for 20 min. After washing with PBS 3 times, DMEM supplemented with 1% FBS was added to each well at 37 °C for the indicated times. For the TEM assay, the above cells were trypsinized and fixed using 2.5% (*v*/*v*) glutaraldehyde fixative solution for 2 h at 4 °C; then, the samples were sent to an electron microscope laboratory for the preparation of thin section and observation of viral and bacterial particles at China Agricultural University.

### 4.4. Lactic Dehydrogenase (LDH) Release Assay

A549 cells were cultured in 96-well plates and treated as indicated above. Then, cells were collected and assessed by a Lactic Dehydrogenase Release Assay Kit (Beyotime) according to the manufacturer’s instructions. The absorbance of each well was measured by a microplate reader (Bio-Rad, Hercules, CA, USA) under 490 nm.

### 4.5. Real-Time Quantitative PCR (RT-qPCR)

Total RNA was extracted from A549 cells or lung tissues using Trizol, and the gene mRNA levels were determined by RT-qPCR as previously described [40,41]. The primers for the genes were as follows: HA: F: 5′-CGCAGTATTCAGAAGAAGCAAGAC-3′; R: 5′-TCCATAAGGATAGACCAGCTACCA-3′.

### 4.6. Virus Titration

TCID50 assays were performed on MDCK cells inoculated with 10-fold serially diluted viruses and incubated at 37 °C for 72 h. TCID_50_ values were calculated according to the Reed–Muench method.

### 4.7. Immunofluorescent Staining

Infected cells were incubated with Mito-Tracker Red CMXRos (Beyotime) for 30 min at 37 °C after the final PBS wash after gentamicin incubation. Then, the Immunol Staining Fix Solution (Beyotime) and Triton X-100 (Beyotime) were added to the cells. Cells were blocked with QuickBlock™ blocking buffer (Beyotime) for 1 h and incubated with appropriate primary antibodies for 12 h at 4 °C, rinsed three times with PBST, and incubated with Alexa Fluor 488-labeled goat anti-rabbit IgG for 1 h at room temperature. Nuclei were stained with DAPI (Beyotime). Images were obtained using a confocal microscope (FV10-ASM, Olympus Microsystems, Tokyo, Japan).

### 4.8. Measurement of ROS Production

A549 cells were digested with EDTA-free trypsin at 12 h and 24 h post-infection, respectively, rinsed with DMEM, and centrifuged at 4 °C, 1000 rpm for 5 min to collect single-cell suspensions. The detection of ROS was performed with a Reactive Oxygen Species Assay Kit (Solarbio) according to the manufacturer’s instructions. The fluorescence signals were recorded using flow cytometry (Olympus, Tokyo, Japan)

### 4.9. Mitochondrial Membrane Potential (MMP) Measurement

The MMP changes were assessed by the Mitochondrial Membrane Potential Assay Kit with JC-1 (Beyotime, China) according to the manufacturer’s instructions. Briefly, cells were washed with PBS 3 times, incubated with 1 mL DMEM with 10% FBS and 1 mL JC-1 staining solution at 37 °C for 20 min, then the supernatant was aspirated and cells were washed twice with JC-1 staining buffer (1X). Subsequently, 2 mL DMEM with 10% FBS was added to the cells and prepared for microscopic observation. JC-1 exists either as a cytoplasmic JC-1 monomer or mitochondrial J-aggregates depending on the potential of the mitochondrial membrane. In healthy cells with high MMP, JC-1 spontaneously forms J-aggregates in mitochondria, which emits red fluorescence. In unhealthy cells, the MMP declines and JC-1 is released from the mitochondria and exists as a monomer in the cytoplasm, which yields green fluorescence.

### 4.10. Rapamycin (Rapa), CCCP, and Mdivi-1 Treatment Model In Vitro

Firstly, CCCP (mitophagy activator (MCE, Shanghai, China)) at CCCP at doses of 5 μM, 10 μM, 15 μM, and 20 μM and Mdivi-1 (mitophagy inhibitor (MCE)) at doses of 5 μM, 10 μM, 20 μM, and 40 μM were selected to determine the optimal doses. Then, A549 cells were pre-treated with Rapa (mTORC1 inhibitor (Beyotime)), CCCP (mitophagy activator (MCE)), and Mdivi-1 (mitophagy inhibitor (MCE)) at 10 μM for 24 h before infection, respectively. Then, cells were infected with IAV and *S. aureus* according to the above procedure.

### 4.11. Western Blot Analysis

A549 cells were harvested by the cell scraper and lysed in RIPA containing 1 mM PMSF (Beyotime) for 15 min on ice. The lysates were centrifuged at 10,000× *g* for 20 min, and the supernatants were collected. BCA Protein Assay Kit (CWBIO, China) was used for quantitative protein detection. Denatured protein samples were separated by SDS gel electrophoresis and electro-transferred onto methanol-activated polyvinylidene difluoride (PVDF) nylon membrane (Millipore, Burlington, MA, USA). Membranes were blocked with 5% blocking buffer for 1.5 h at room temperature (nonfat powdered milk dissolved in TBST (Solarbio)). The membranes were then incubated with appropriate primary antibodies overnight at 4 °C in a shaking incubator. On the following day, the membranes were washed with TBST 3 times (10 min/time) and then incubated with HRP-conjugated secondary antibodies for 1.5 h at room temperature. After washing with TBST 3 times, proteins were detected using a Clarity Western ECL Substrate (Bio-Rad) and chemiluminescence gel imaging system (Tanon-5200Multi). β-actin served as a loading control. ImageJ software (v1.53t) was used to analyze the gray degree values of the protein bands.

### 4.12. Intracellular Bacteria Colony Counting

Infected cells were incubated with gentamicin for 20 min and lysed by 500 μL 0.1% Triton X-100. The lysates were diluted continuously by sterile PBS, and each dilution was inoculated onto three BHI agar plates per dilution. The plates were cultured in a biochemical incubator at 37 °C. After 16–24 h of incubation, the number of colonies of each agar plate was counted.

### 4.13. Terminal-Deoxynucleoitidyl Transferase Mediated Nick End Labeling (TUNEL)

The presence of apoptotic cells was determined via the TUNEL assay, which was performed using an In Situ Cell Death Detection Kit in accordance with the manufacturer’s instructions (Roche, Philadelphia, PA, USA). The slides were examined under a laser scanning confocal microscope (Leica TCS SP5 II, Leica Microsystems, Wetzlar, Germany).

### 4.14. Infection of Mice In Vivo

This study followed the ARRIVE guidelines. The 7-week-old specific pathogen-free (SPF) BALB/c female mice were purchased from Beijing Vital River Laboratory Animal Technology (Beijing, China). Mice were housed in independent ventilated cages and received pathogen-free food and water. Forty mice were randomly divided into four groups: PBS group, IAV group, IAV + *S. aureus* group, and *S. aureus* group. Mice were anesthetized with Zoletil^®^ (Virbac, Carros, France) and sequentially infected with 100 PFU/mouse H1N1 virus (in 50 μL sterile PBS) intranasally or PBS alone for 5 days, followed by infection with 5 × 10^7^ CFU/mouse (in 50 μL sterile PBS) MRSA intranasally [42]. The mice were observed daily for a further 14 days, and the clinical symptoms, the changes in body weight, and survival rates were recorded. All experiments were approved by the Animal Ethics Committee of China Agricultural University.

### 4.15. CCCP and Mdivi-1 Treatment Model In Vivo

Thirty-six 7-week-old SPF BALB/c female mice were randomly divided into six groups: DMSO group, CCCP group, Mdivi-1 group, IAV + *S. aureus* + DMSO group, IAV + *S. aureus* + CCCP group, and IAV + *S. aureus* + Mdivi-1 group. Mice were abdominally injected with CCCP (5 mg/kg) and Mdivi-1 (25 mg/kg) for two days before infection, respectively. Then, mice were infected with IAV and *S. aureus* according to the above procedure. At 6 days post-infection, the clinical symptoms of mice and gross lesions of the lungs were observed. The pathological changes in the lungs of mice were scored according to the following scale: 0 = no clinical signs; 1 = mild signs (slight swelling, small percentage of hemorrhage area and wet surface); 2 = moderate signs (moderate swelling, moderate percentage of hemorrhage area and wet surface); 3 = severe signs (severe swelling, large percentage of hemorrhage area and wet surface); 4 = extremely severe signs (extremely severe swelling, entire hemorrhage area and wet surface).

### 4.16. Histology and Immunochemistry

At 6 days post-infection, the lungs of mice were collected and fixed in 10% formalin. Histopathological changes in the lung tissues were evaluated using hematoxylin and eosin (H&E) staining [43]. Histopathological scores were carried according to the following scoring criteria: 0 = no microscopic lesions; 1 = extremely mild, characterized by hemorrhage and hyperemia; 2 = mild, characterized by hemorrhage and hyperemia, perivascular edema and mild infiltration of inflammatory cells; 3 = moderate, characterized by hemorrhage and hyperemia, perivascular inflammation, and thickening of the pulmonary interstitium; 4 = severe, characterized by serious pulmonary structural damage, disappearance of alveoli, hyperemia and greater infiltration of inflammatory cells and severe desquamation of epithelial cells. Examination of influenza viral antigens in the lungs was performed by immunohistochemical (IHC) staining using an anti-influenza virus nucleoprotein monoclonal antibody at 1:1000 dilution [43]. The number of positive cells was counted and analyzed. All sections were examined under light microscopy (CX31, Olympus, Tokyo, Japan).

### 4.17. Bacterial Load Detection of Lungs

The bacterial load in the lungs was qualified by colony counting. Briefly, 50 mg of lung tissue was collected and homogenized. The homogenate was diluted in a continuous gradient with PBS, and then 100 μL of the diluted homogenate was inoculated on three BHI agar plates per dilution. The plates were cultured in a biochemical incubator at 37 °C. After 18 h of incubation, the number of colonies of each agar plate was counted.

### 4.18. Statistical Analysis

The data were expressed as mean ± SD. The two-way ANOVA with Tukey’s multiple comparisons test with GraphPad Prism (version 8.0) was used to determine the significance between groups. *p* < 0.05 was considered statistical significance (* *p* < 0.05, ** *p* < 0.01, *** *p* < 0.001, **** *p* < 0.0001).

## Data Availability

All data generated or analyzed during this study are included in this published article.

## Supplementary Materials

The following supporting information can be downloaded at: https://www.mdpi.com/article/10.3390/ijms26094162/s1. Figure S1: IAV and *S. aureus* particles in A549 cells at 24 h post-infection following IAV and secondary *S. aureus* infection by TEM. Figure S2: The Mdivi-1 and CCCP at optimal doses were selected for subsequent experiments. Figure S3: Effects of mitophagy on the lung injury in mice following IAV and secondary *S. aureus* infection.
