# Supplementary material for: Vital Role of PINK1/Parkin-Mediated Mitophagy of Pulmonary Epithelial Cells in Severe Pneumonia Induced by IAV and Secondary Staphylococcus aureus Infection
Source: Int J Mol Sci. 2025 Apr 27;26(9):4162. doi: 10.3390/ijms26094162 (PMC12071998; doi:10.3390/ijms26094162)
Supplement: Supplementary file 1 [file ijms-26-04162-s001.zip › ijms-3566020-supplementary.pdf]

Supplementary Materials

Vital role of PINK1/Parkin-mediated mitophagy of pulmonary epithelial cells in severe pneumonia induced by IAV and secondary *Staphylococcus aureus* infection

Caiyun Huo[1†], Yuli Li[1†], Yuling Tang[1], Ruijing Su[1], Jiawei Xu[1], Hong Dong[2], Yanxin Hu[1*], Hanchun Yang[1]

[1] Key Laboratory of Animal Epidemiology of Ministry of Agriculture and Rural Affairs, National Key Laboratory of Veterinary Public Health and Safety, College of Veterinary Medicine, China Agricultural University, Beijing, China.
[2]Beijing Key Laboratory of Traditional Chinese Veterinary Medicine, Beijing University of Agriculture, Beijing, China.

† Contributed to the work equally.

*** Corresponding author:**
Dr Yanxin Hu, National Key Laboratory of Veterinary Public Health and Safety, Key Laboratory of Animal Epidemiology of Ministry of Agriculture and Rural Affairs, College of Veterinary Medicine, China Agricultural University, Beijing, China; Phone: +86 010 62731977; Email: huyx@cau.edu.cn

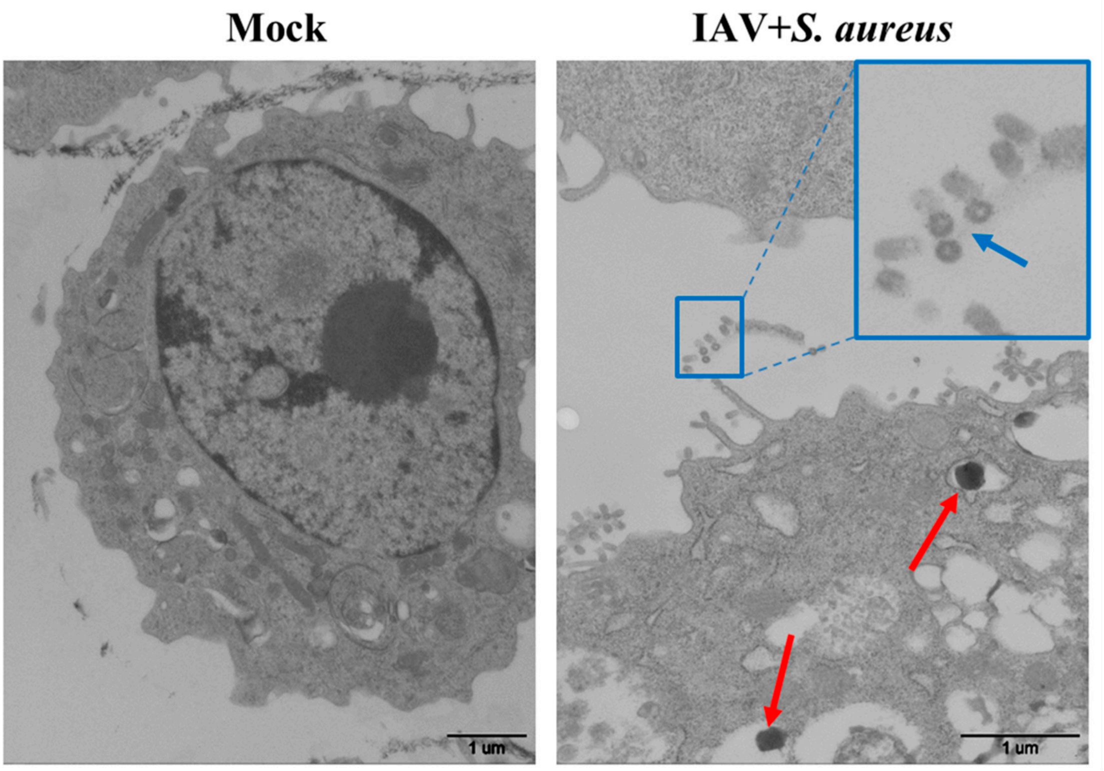

Figure. S1 IAV and *S. aureus* particles in A549 cells at 24 h post-infection following IAV and secondary *S. aureus* infection by TEM. Red arrows indicated *S. aureus* and blue arrows indicated virions.

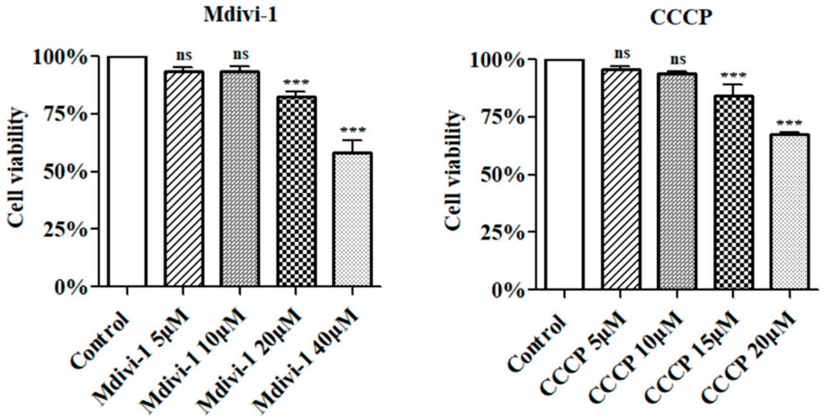

Figure. S2 The Mdivi-1 and CCCP at optimal doses were selected for subsequent experiments. A549 cells were treated with CCCP at doses of 5 μM, 10 μM, 15 μM, 20 μM and Mdivi-1 (mitophagy inhibitor (MCE, China)) at doses of 5 μM, 10 μM, 20 μM, 40 μM for 24 h, respectively. Then, cell viability was detected. ***P<0.001.

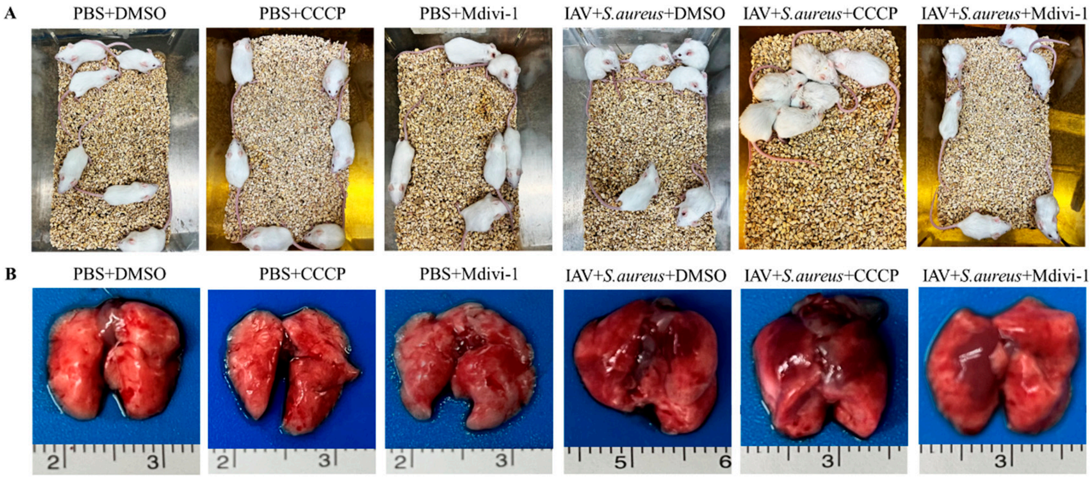

Figure. S3 Effects of mitophagy on the lung injury in mice following IAV and secondary *S. aureus* infection. Mice were abdominally injected with CCCP (5 mg/kg) and Mdivi-1 (25 mg/kg) for two days before infection. After infection of IAV and secondary *S. aureus*, lung tissues were collected at 6 days post-infection. (A-B) The clinical symptoms of mice and gross lesions of lungs were observed (n=3).
